# Three Alternative Protocols for the Refabrication of Prosthesis: An Effective Way to Use the Current Prosthesis

**DOI:** 10.7759/cureus.85393

**Published:** 2025-06-05

**Authors:** Yuka Sumita, Mahmoud E Elbashti, Hiroko Tani, Yusuke Mano, Mariko Hattori, Noriyuki Wakabayashi

**Affiliations:** 1 Department of Partial and Complete Denture, The Nippon Dental University School of Life Dentistry at Tokyo, Tokyo, JPN; 2 Department of Advanced Prosthodontics, Institute of Science Tokyo, Tokyo, JPN; 3 Faculty of Dentistry, University of Zawia, Zawia, LBY; 4 Dental Laboratory, Institute of Science Tokyo Hospital, Tokyo, JPN

**Keywords:** custom tray, digitization, exiting prosthesis, fabrication procedure, refabrication

## Abstract

The effective use of a patient’s existing prosthesis can be an alternative to creating a new prosthesis and is faster, simpler, and less stressful for clinicians and patients. This report describes three minimally burdensome protocols for refabricating a maxillofacial prosthesis already in use. It also explains how to use the impression of the existing prosthesis to create a new one. Three protocols were introduced to refabricate a new prosthesis skipping the step of preliminary impression: (1) a putty impression protocol, where an impression of the intaglio surface of the existing prosthesis was made using heavy body polyvinyl siloxane impression material and prepared as a working cast; (2) an irreversible hydrocolloid impression protocol, where an impression of the surface of the existing prosthesis was made using hydrocolloid impression material and a duplicating flask; and (3) a digitized protocol, where an intraoral scanner was used to duplicate the existing maxillary prosthesis and print it using a 3D printer. All three protocols can help effectively skip the step of preliminary impression using a stock tray. The benefits include the avoidance of chair time in the preliminary impression step. Also, the custom tray or the copy denture made from the impression of the existing prosthesis is helpful because it reflects the morphology of a prosthesis that has been adjusted in multiple steps over a long period. These protocols can be used for both maxillofacial prostheses and complete dentures.

## Introduction

Maxillofacial prostheses are used in patients with a maxillofacial defect following surgery [1] to help them with speaking [2], swallowing [3], and mastication [4] and to achieve optimal aesthetic outcomes [5]. Creation of a maxillofacial prosthesis is complicated, not only because of the nature of the bone defect but also the movement of the surrounding soft tissue (e.g., grafted skin), which is prone to bleeding and at risk of material impaction, trismus, and other unfavorable events [1]. Fabrication of a maxillofacial prosthesis is even more challenging in edentulous patients with defects, given the lack of structures for support and retention. The process of recording jaw relation can often lead to errors, even if we apply a specific technique such as a base plate [6]. During rehabilitation, these patients may need refabrication of the prosthesis they are currently using, especially if it is worn, broken, or damaged due to repeated adjustments.

With conventional methods, the steps used to create a second prosthesis are the same as those used for primary fabrication, beginning with a stock tray [1]. However, this method is often inefficient because it requires repeating all the complicated steps to make a maxillofacial prosthesis. Using impression material to duplicate prostheses is one option [7,8]. In a previous report, Elbashti et al. proposed a method for duplicating an old existing prosthesis using cone-beam CT [9]. With the advent of digital technology, we now have more options for duplicating a prosthesis [10,11]. The clinical protocols introduced in this report can be performed rapidly and are less burdensome since they entail refabricating a maxillary prosthesis already in use.

## Technical report

The protocols

The three protocols described below were used to refabricate the existing maxillary prosthesis.

1. Putty Impression Protocol

**Figure 1:**
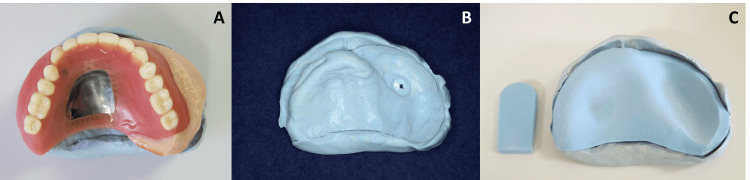
Putty impression protocol A: making an impression of the existing prosthesis; B: the obtained impression of the intaglio surface of the prosthesis; C: fabricated custom-made impression tray on the putty model

An impression of the intaglio surface of the existing prosthesis was made using a heavy body polyvinyl siloxane impression material (Exafine Putty Type, GC, Tokyo, Japan) and was prepared as a putty model. Then, the acrylic tray material (Ostron II, GC) was applied to the putty model to prepare a custom-made impression tray without a handle. A handle was prepared separately (Figure 1). The tray was placed in the oral cavity and checked for fit. The handle was attached to the tray using acrylic resin, being mindful of the occlusal plane [10]. The final impression was made using the tray and a regular type polyvinyl siloxane impression material (Exafine Regular and Injection, GC), and the prosthesis fabrication was completed as usual.

2. Irreversible Hydrocolloid Impression Protocol

**Figure 2:**
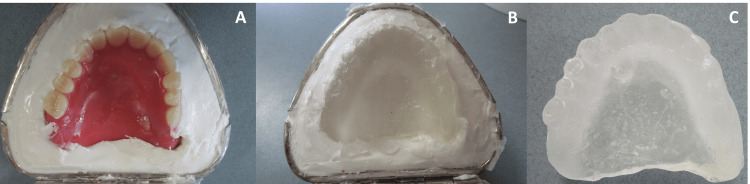
Irreversible hydrocolloid impression protocol A: making an impression of the existing prosthesis; B: fabricating a copy denture in a duplicating flask; C: the completed copy denture

An impression of the surface of the existing prosthesis was made using irreversible hydrocolloid impression material (Algiace Z, Dentsply Sirona, Tokyo, Japan) in a duplicating flask. A self-polymerizing acrylic resin (Palapress Vario, Heraeus, Hanau, Germany) was poured, and the copy denture was fabricated as a custom-made impression tray without a handle (Figure 2). The copy denture was then placed in the oral cavity and checked for fit. The final impression was made with the polyvinyl siloxane impression material using the copy denture as an individual tray. At the same time, the lip support and the occlusal relationship were checked and recorded, and the prosthesis fabrication was completed as usual.

3. Digitized Protocol

**Figure 3:**
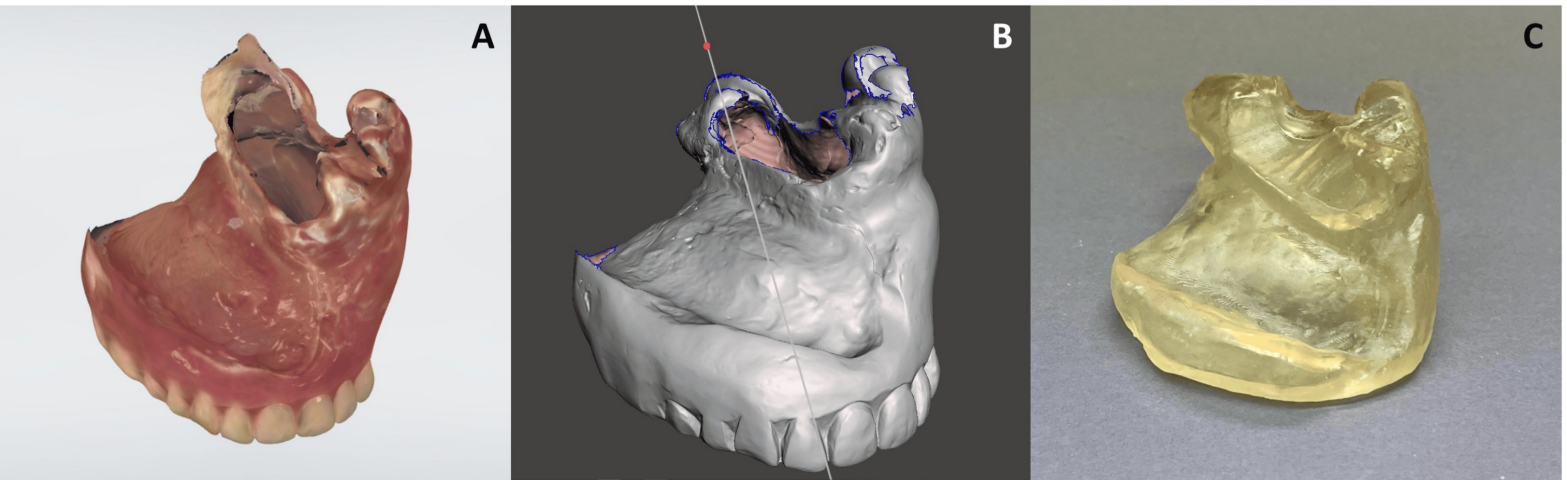
Digitized protocol A: scanned digital data for an existing maxillary denture; B: the designed copy denture for printing out; C: the three-dimensional printed copy denture

An intraoral scanner (Trios 3, 3Shape, Copenhagen, Denmark) was used to make a digital impression of the surface of the existing prosthesis (Figure 3A). A copy denture was designed (Figure 3B) and fabricated by 3D printing material (DentalSG, Formlabs, Somerville, MA) with a 3D printer (Form3, Formlabs) (Figure 3C). The copy denture was placed in the oral cavity and checked for fit. The final impression was made using the 3D-printed copy denture and the polyvinyl siloxane impression material. At the same time, the lip support and the occlusal relationship were checked and recorded. Then, the prosthesis fabrication was completed as usual. The use of a model scanner, such as a desktop lab scanner (D2000 Lab Scanner, 3Shape), is another option for this protocol.

## Discussion

Problems with speech, [2] swallowing, [3] and mastication [4] stemming from insufficient support and retention of a maxillofacial prosthesis are major concerns in patients with a maxillary defect. The prosthesis must fill the unique denture space in patients with mandibular and/or tongue defects. Whether or not a maxillary or mandibular prosthesis is used, the fabrication of a maxillofacial prosthesis is usually completed after making many adjustments over a long period. Therefore, the patient’s existing prosthesis contains a wealth of information because its unique structure is decided by the movement of the lips, cheeks, and tongue over time. However, the presence of bone defects as well as reconstruction using bone and skin flaps makes the structure of the oral cavity especially complicated. For these patients, the stock tray must be modified by cutting or adding material such as impression compound. Therefore, a longer chair time is required, increasing the burden on patients and dentists.

When using conventional fabrication methods, the stock tray is used for the primary impression as well as for a second prosthesis [1]. However, patients with a maxillofacial defect often have trismus, which makes tray insertion difficult. Furthermore, the shape of a stock tray usually does not fit their oral cavity. The three protocols described are helpful for refabricating prostheses for patients with a maxillofacial defect and for refabricating complete dentures for patients with a normal jaw. Utilizing the current denture structure can help dentists perform refabrication efficiently.

The protocols described here can skip the preliminary impression and allow fast and simple refabrication of the maxillary prosthesis that the patient is already using. In protocol 1, heavy body polyvinyl siloxane is a material familiar to dentists and helps replicate the structure of the existing prosthesis. The benefit of protocol 1 is that it does not require any special devices. However, protocol 2 is a reasonable choice if there is an instrument for refabricating the copy denture. Protocol 3 can be selected if the facility has an intraoral scanner or model scanner and access to a 3D printer. Occasionally, it is possible to make a copy denture as a definitive prosthesis in protocol 3. The critical point is that each prosthodontist can select a reasonable protocol according to the devices available and the patient’s preferred prosthesis design [10,11]. Therefore, the aim of this report is not to determine which protocol is the best. Still, the question of which method yields the most accurate results remains of interest, and future studies may explore comparative accuracy to further inform clinical decision-making.

Protocol 1 introduces the chairside addition of the tray handle. Recording the occlusal plane is important because the remaining jaw is often asymmetric in patients with a maxillofacial defect [12]. This can cause problems in communicating such information between dental technicians and dentists. However, because the direction of the handle added at the chairside is parallel to the occlusal plane, which helps dental technicians recognize this plane, it makes subsequent steps easier and more efficient to complete [12]. Using the handle is, therefore, expected to be beneficial. Protocols 2 and 3 allow us to make a record of the occlusal relationship at the same time [10,11]. The scope of application of these three protocols extends to all complete dentures. In cases involving mandibullectomy patients or those with severely resorbed alveolar ridges, it can be difficult to achieve a proper fit, often requiring multiple adjustments or relining procedures to attain stability. In such situations, these protocols can be useful for fabricating a new prosthesis. The use of these protocols is not limited to maxillofacial prostheses.

## Conclusions

The three protocols described here are effective for fabricating a custom tray or a copy maxillary prosthesis. One major benefit is the avoidance of chair time in the preliminary impression step. Moreover, the individual tray or copied prosthesis made from the impression of the existing prosthesis is useful because it reflects the morphology of the prosthesis, which has been adjusted in multiple steps over a long period.
